# Cross-Sectional Assessment of Nut Consumption and Obesity, Metabolic Syndrome and Other Cardiometabolic Risk Factors: The PREDIMED Study

**DOI:** 10.1371/journal.pone.0057367

**Published:** 2013-02-27

**Authors:** Núria Ibarrola-Jurado, Mònica Bulló, Marta Guasch-Ferré, Emilio Ros, Miguel A. Martínez-González, Dolores Corella, Miquel Fiol, Julia Wärnberg, Ramón Estruch, Pilar Román, Fernando Arós, Ernest Vinyoles, Lluis Serra-Majem, Xavier Pintó, María-Isabel Covas, Josep Basora, Jordi Salas-Salvadó, the PREDIMED Study Investigators

**Affiliations:** 1 Human Nutrition Unit, Hospital Universitari de Sant Joan de Reus, Institut d‘Investigació Sanitària Pere Virgili (IISPV), Universitat Rovira i Virgili, Reus, Spain; 2 CIBER Fisiopatología de la Obesidad y Nutrición (CIBERobn), Instituto de Salud Carlos III (ISCIII), Madrid, Spain; 3 Lipid Clinic, Endocrinology and Nutrition Service, Hospital Clinic, Institut d’Investigacions Biomèdiques August Pi Sunyer (IDIBAPS), Barcelona, Spain; 4 Department of Preventive Medicine and Public Health, Medical School-Clinica, University of Navarra, Pamplona, Spain; 5 Department of Preventive Medicine and Public Health, University of Valencia, Valencia, Spain; 6 University Institute for Health Sciences Investigation, Palma de Mallorca, Spain; 7 Department of Preventive Medicine, University of Málaga, Málaga, Spain; 8 Department of Internal Medicine, Hospital Clinic, Institut d’Investigacions Biomèdiques August Pi Sunyer (IDIBAPS), Barcelona, Spain; 9 Department of Family Medicine, Primary Care Division of Sevilla, Mercedes Navarro Health Center, Sevilla, Spain; 10 Clinical Trial Unit, Hospital Txangorritxu, Vitoria, Spain; 11 La Mina Primary Care Center, University of Barcelona, IDIAP Jordi Gol. Barcelona, Spain; 12 Department of Clinical Sciences, University of Las Palmas de Gran Canaria, Las Palmas, Spain; 13 Internal Medicine Service, Hospital of Bellvitge, Hospitalet de Llobregat, Spain; 14 Cardiovascular Risk and Nutrition Research Group, IMIM-Institut de Recerca del Hospital del Mar, Barcelona, Spain; University of Las Palmas de Gran Canaria, Spain

## Abstract

**Introduction:**

Prospective studies have consistently suggested that nut consumption is inversely related to fatal and non-fatal coronary heart disease. Limited data are available on the epidemiological associations between nut intake and cardiometabolic risk factors.

**Objective:**

To evaluate associations between frequency of nut consumption and prevalence of cardiometabolic risk factors [obesity, metabolic syndrome (MetS), type-2 diabetes, hypertension, and dyslipidemia] in a Mediterranean population at high cardiovascular risk.

**Materials and Methods:**

Cross-sectional study of 7,210 men and women (mean age, 67 y) recruited into the PREDIMED study. MetS was defined by the harmonized ATPIII and IDF criteria. Diabetes and hypertension were assessed by clinical diagnosis and dyslipidemia (high triglycerides, low HDL-cholesterol, and hypercholesterolemia) by lipid analyses. Nut consumption was assessed using a validated food frequency questionnaire and categorized as <1, 1–3, and >3 servings/wk. Control of confounding was done with multivariate logistic regression.

**Results:**

Compared to participants consuming <1 serving/wk of nuts, those consuming >3 servings/wk had lower adjusted odds ratios (OR) for obesity (0.61, 95% confidence interval 0.54 to 0.68; P-trend <0.001), MetS (0.74, 0.65 to 0.85; P-trend<0.001), and diabetes (0.87, 0.78 to 0.99; P-trend = 0.043). Higher nut consumption was also associated with lower risk of the abdominal obesity MetS criterion (OR 0.68, 0.60 to 0.79; P-trend<0.001). No significant associations were observed for the MetS components high blood pressure, dyslipidemia, or elevated fasting glucose.

**Conclusions:**

Nut consumption was inversely associated with the prevalence of general obesity, central obesity, MetS, and diabetes in subjects at high cardiovascular risk.

## Introduction

The worldwide prevalence of obesity, the metabolic syndrome (MetS), and associated comorbidities with a high risk of disability and death, such as type-2 diabetes, hypertension and cardiovascular disease (CVD), is steadily increasing and has become a major public health problem. The MetS, composed of abdominal obesity associated with insulin resistance, fasting hyperglycemia or frank diabetes, high triglycerides, low HDL-cholesterol (HDL-C), and high blood pressure, is a paradigm of increased risk for CVD [1]. MetS roughly affects one out of each three adults in the world population [2], but this is a conservative estimate based on the rather high waist circumference thresholds of the National Cholesterol Education Program Adult Treatment Panel III definition [1] and would certainly be higher if recently defined ethnic-specific cutoffs for abdominal obesity were used [3].

With the rapidly rising prevalence of MetS and diabetes, there is an urgent need to identify preventive lifestyle strategies. As recently reviewed [4], landmark clinical trials of lifestyle changes in overweight or obesity subjects with prediabetes, most of whom had MetS, have shown that diet and exercise leading to weight loss consistently reduce cardiometabolic risk factors and the incidence of diabetes. There is also epidemiological and clinical trial evidence supporting that increased adherence to the Mediterranean diet (MedDiet) [5] and consumption of foods rich in antioxidants, n-3 fatty acids, or minerals other than sodium [6] relates to a reduced prevalence of the MetS.

Based on their nutrient profile and known effects on heart health [7], nuts are a unique food that could be useful to improve cardiometabolic risk factors, the MetS and diabetes. Nuts are already considered as a key component of a cardioprotective diet [8]. In fact, nuts were the first whole food that was granted a heart health claim by the US Food and Drug Administration [9]. Nuts are a rich source of energy because 45 to 75% of their weight is made up of fat, but this fat is mostly unsaturated [10]. Nuts also contain high-quality protein, fiber, antioxidant vitamins, and minerals such as magnesium, copper, selenium, potassium, and little sodium except when salted [11], [12]. Other bioactive compounds that abound in nuts are phytosterols [12] and polyphenols, particularly flavanoids and proanthocyanidins [13], [14].

While prospective studies have shown a consistent association between increased nut consumption and a reduced risk of coronary heart disease [7], the epidemiological evidence for a beneficial effect on weight gain or obesity [15], the risk of MetS and diabetes [16], or hypertension incidence [17] is less clear. Also, clinical trials with different types of nuts, including peanuts (technically a legume, but included in the nut group because of a similar nutrient profile), have consistently demonstrated that regular consumption has a hypocholesterolemic effect [7], [18]. There is also suggestive evidence that nut consumption improves oxidation [19], inflammation [20], and endothelial function [17], while no clear effect on blood pressure has been detected thus far. On the other hand, data on nut consumption and glycemic control or insulin sensitivity are inconclusive [16].

Given these uncertainties, it is important to assess associations between nut consumption and cardiometabolic risk factors in a large sample of individuals with a wide range of nut intake. Such is the cohort of participants in the PREDIMED study, a large multicenter dietary intervention trial for the primary prevention of CVD [21]. Therefore, in a cross-sectional assessment, we examined the associations between frequency of nut intake and prevalence of cardiovascular risk factors in subjects at high cardiovascular risk recruited into the PREDIMED trial.

## Materials and Methods

### Study Population

This cross-sectional analysis was conducted with the baseline data of participants in the PREDIMED study (PREvención con DIeta MEDiterránea), a large, parallel-group, multicenter, randomized, controlled clinical trial aimed to assess the effects of the MedDiet on the primary prevention of CVD (www.predimed.es and www.predimed.org). The design of the PREDIMED trial has been reported in detail elsewhere [21]. A total of 7,447 participants, aged between 55–80 y in men and 60–80 y in women were recruited between October 2003 and January 2009. Eligible participants were free of CVD at baseline, and had either type-2 diabetes or at least 3 of the following cardiovascular risk factors: current smoking, body mass index (BMI) ≥25 kg/m^2^; blood pressure ≥140/90 mmHg or treatment with antihypertensive medication; serum LDL-cholesterol (LDL-C) ≥160 mg/dL or treatment with lipid-lowering drugs; HDL-cholesterol (HDL-C) ≤40 mg/dL in men and ≤50 mg/dL in women; or family history of early-onset CVD (≤55 years in men and ≤60 years in women). Exclusion criteria included any severe chronic illness, alcohol or drug abuse, and BMI ≥40 kg/m^2^. The local institutional review boards approved the protocol and all participants provided written informed consent.

### Nut Consumption

In the present study peanuts, almonds, hazelnuts, walnuts, pine nuts, pistachios, macadamia and cashews were all considered nuts. A 137-item food frequency questionnaire (FFQ) was used to determine food consumption. Detailed information about the development of the FFQ and its reproducibility and validity in the PREDIMED cohort has been previously reported [22]. Twenty eight grams of nuts was considered one serving.

### Adherence to the Mediterranean Diet

To assess adherence to the MedDiet, a 14-item questionnaire with a value of 0 or 1 for each dietary component was used [21]. Each item refers to a characteristic feature of the MedDiet, for example: amount and use of olive oil for cooking and dressing; weekly intake of nuts; increased consumption of vegetables, fruits, legumes and fish; recommended intake of white meat instead of red or processed red meat, moderate wine consumption; avoid eating butter, fast-food, sweets, pastries or sugar-sweetened beverages; to dress dishes by typical “sofrito” sauce (using tomato, garlic, onion and spices with olive oil). For the purpose of controlling potential confounding by the overall dietary pattern we used this score as a covariate in multivariable models. Therefore, in this study the question on nut consumption was removed and only a 13-point score was considered (minimum score = 0, maximum score = 13).

### Metabolic Syndrome

MetS was defined according to the updated joint criteria of the International Diabetes Federation (IDF) and the American Heart Association/National Heart, Lung, and Blood Institute [3]. Participants received a diagnosis of MetS if they presented at least 3 of the followings 5 components: 1) elevated waist circumference (≥102 cm for men and ≥88 cm for women); 2) elevated serum triglycerides (≥150 mg/dL) or triglyceride-lowering medication use; 3) reduced HDL-C (<40 mg/dL in men and <50 mg/dL in women); 4) elevated blood pressure (≥130/85 mm Hg) or use of antihypertensive medication; and 5) high fasting glucose (≥100 mg/dL) or hypoglycaemic drug treatment.

### Assessment of Other Outcomes

Obesity was defined as BMI ≥30 kg/m^2^. Diabetes and hypertension were considered to be present by clinical diagnosis and/or use of antidiabetic or antihypertensive medication, respectively. Atherogenic dyslipidemia was defined by the association of serum triglycerides ≥150 mg/dL and HDL-C <40 mg/dL in men or <50 mg/dL in women. Hypercholesterolemia was considered when LDL-C was ≥130 mg/dL.

### Procedures

We administered a general questionnaire about lifestyle, including smoking habits, socio-demographics conditions, and history of illnesses and medication use. A validated Spanish version of the Minnesota Leisure Time Physical Activity Questionnaire was also administered. Trained personnel measured weight (kg) and height (cm) with calibrated scales and a wall-mounted stadiometer, respectively, and waist circumference with an anthropometric tape midway between the lower rib and the superior border of the iliac crest. BMI was calculated as weight in kilograms divided by the square of height in meters. Blood pressure was measured in triplicate with 5-min intervals between each measurement with the subject in a sitting position by using a validated semiautomatic oscillometer (Omron HEM-705CP, Hoofddorp, The Netherlands). After an overnight fast, samples of serum, EDTA plasma and urine were obtained and stored at −80°C until analysis. Serum glucose, triglycerides and cholesterol concentrations were determined by standard enzymatic methods in automatic autoanalyzers.

### Statistical Analyses

Results are expressed as mean ± SD or percentages. We divided participants into three categories of nut consumption at baseline (less than one serving/wk, 1–3 servings/wk, and more than 3 servings/wk). Participants with extremes of total energy intakes (<500 or >3500 kcal/day in women, and <400 or >4000 kcal/day in men) were excluded. We also excluded participants reporting nut intake greater than 100 g/d.

To compare quantitative and categorical variables between categories of nut consumption, ANOVA and chi-square tests were used, respectively. We used multiple logistic regression models to examine the associations of the prevalence of different outcomes across categories of nut consumption. Results are expressed as odds ratios (OR) and 95% confidence intervals (CI). Regressions models were adjusted for potential confounding factors using three different models to allow for different levels of adjustment. The first model was adjusted for age, sex, geographic recruitment area and BMI (kg/m^2^); the second model was additionally adjusted for smoking status (no smoker, former and current), leisure time physical activity (MET-min/day), and education level (primary or illiterate, secondary and university); the third model was additionally adjusted for energy consumption (kcal/d) and adherence to the Mediterranean diet (13-point score). Obesity, abdominal obesity and metabolic syndrome were not adjusted for BMI because of collinearity among these variables. Hypercholesterolemia was additionally adjusted by statin treatment (yes/no). Analyses were performed by using the SPSS statistical package version 19.0 (SPSS Inc., Chicago, IL) and the level of significance was set at p<0.05.

## Results

After excluding participants with total energy intake out of predefined limits (n = 153) or implausible values for nut consumption (n = 6), 7,210 participants remained for the present analysis. There were a total of 3,067 men and 4,143 women with mean age 67 y. Average nut consumption in each of the 3 categories was 0.48±0.86, 5.88±1.82 and 25.48±13.23 g/d, respectively. The general characteristics of participants are summarized in **Table 1**. Compared to individuals consuming less than one serving of nuts per wk, those consuming more than 3 servings per wk were younger and more physically active and had a lower BMI and waist circumference, and a higher adherence to the MedDiet. In addition the percentage of current smokers was lower and the prevalence of individuals with high education level was higher in the highest category of nut consumption. A lower percentage of subjects in this category were obese or had diabetes. **Table 2** shows similar data for MetS, its components, and atherogenic dyslipidemia. The prevalence of MetS, abdominal obesity, and atherogenic dyslipidemia decreased across categories of nut intake, while participants consuming >3 servings/wk showed a lower prevalence of high triglycerides, low HDL-C, and high fasting glucose compared with those consuming <1 serving/wk. On the other hand, the prevalence of hypertension was unrelated to frequency of nut consumption.

**Table 1:** General characteristics of the study population by servings/week of nuts.

	Nut consumption			
	<1 serving/week(n = 2796)	1–3 servings/week(n = 2125)	>3 servings/week(n = 2289)	P value^1^
**Nut intake (g/day)**	0.48±0.86	5.88±1.82	25.48±13.23	
**Age (years)**	67.4±6.3	66.6±6.2	67.0±6.2	<0.001
**Women, % (n)**	62.2 (1740)	56.0 (1189)	53.0 (1214)	<0.001
**BMI (kg/m** 2 **)**	30.4±4.0	29.9±3.8	29.4±3.7	<0.001
**Weight (kg)**	77.2±12.0	77.2±12.2	75.9±11.6	<0.001
**Waist circumference (cm)**	101.2±10.5	100.9±10.4	99.2±10.0	<0.001
**Leisure-time energy expenditure** **in physical activity (MET-min/day)**	201.2±218.5	234.2±238.4	264.1±257.2	<0.001
**Smoking status, % (n)**				<0.001
** Never**	63.6 (1779)	61.0 (1296)	59.4 (1360)	
** Current**	14.7 (411)	14.2 (301)	12.7 (291)	
** Former**	21.7 (606)	24.8 (528)	27.9 (638)	
**Marital status (% married) (n)**	72.9 (2035)	78.1 (1658)	78.8 (1804)	<0.001
**Education level, % (n)**				<0.001
** Illiterate/primary education**	81.6 (2281)	74.8 (1590)	75.6 (1730)	
** Secondary education**	13.1 (366)	16.5 (351)	16.2 (377)	
** Academic/graduate**	5.3 (149)	8.7 (184)	8.0 (182)	
**Mediterranean diet adherence (13-point score)**	8.1 (1.8)	8.3 (1.8)	8.6 (1.8)	<0.001
**Obesity (BMI ≥30 kg/m** 2 **), % (n)**	53.1 (1486)	46.4 (987)	39.1 (895)	<0.001
**Diabetes, % (n)**	51.4 (1438)	47.6 (1011)	46.2 (1057)	<0.001
**Hypertension, % (n)** 2	83.9 (2346)	81.7 (1737)	82.3 (1883)	0.108
**Hypercholesterolemia (LDL-C >130 mg/dl), % (n)**	47.7 (1201)	49.3 (964)	48.9 (1035)	0.528
**Medication use, % (n)**				
** Oral antidiabetic drugs**	34.7 (968)	31.8 (676)	29.7 (678)	<0.001
** Insulin**	8.9 (247)	6.0 (128)	5.3 (120)	<0.001
** Antihypertensive drugs**	75.6 (2108)	71.4 (1516)	71.1 (1621)	0.001
** Statins**	40.8 (1141)	39.8 (846)	39.8 (912)	0.710
** Fibrates**	3.5 (97)	4.1 (88)	4.2 (96)	0.326

Data are mean ± SD. Abbreviations: BMI: body mass index, LDL-C: low-density lipoproteins cholesterol.

1 ANOVA or chi-square test as appropriate.

2 Defined by medical diagnosis.

**Table 2:** Prevalence of metabolic syndrome, its components, and atherogenic dyslipidemia by servings/week of nuts.

	Nut consumption			
	<1 serving/week(n = 2796)	1–3 servings/week(n = 2125)	>3 servings/week(n = 2289)	P value^1^
**Metabolic syndrome, % (n)**	69.7 (1770)	64.3 (1266)	59.9 (1251)	<0.001
** Abdominal obesity, % (n)**	77.6 (2089)	74.4 (1546)	66.7 (1487)	<0.001
** Hypertriglyceridemia, % (n)**	33.1 (842)	29.5 (578)	30.0 (635)	0.014
** Low HDL-cholesterol, % (n)**	33.2 (841)	31.2 (613)	28.3 (602)	0.001
** High blood pressure, % (n)** 2	94.3 (2625)	93.7 (1984)	94.7 (2155)	0.356
** Fasting plasma glucose** **≥100 mg/dL, % (n)**	69.7 (1784)	64.1 (1267)	65.8 (1365)	<0.001
**Atherogenic dyslipidemia, % (n)** 3	14.9 (378)	12.5 (244)	11.5 (245)	0.002

1 Chi-square test.

2 Defined as blood pressure ≥130/85 mmHg or antihypertensive drug treatment.

3 Defined as serum triglycerides ≥150 mg/dL associated with HDL-cholesterol <40 mg/dL in men or <50 mg/dL in women.

**Table 3** shows the multivariable-adjusted ORs for the main outcomes by categories of nut consumption. In fully-adjusted models, participants in the upper category of nut consumption had a lower prevalence of obesity (OR = 0.61, 95% CI 0.54 to 0.68; P-trend <0.001), diabetes (OR = 0.87, 0.78 to 0.99; P-trend = 0.043) and MetS (OR = 0.74, 0.65 to 0.85; P-trend <0.001) compared with those in the lowest category. No associations were detected for hypertension, atherogenic dyslipidemia, or hypercholesterolemia. **Table 4** depicts adjusted ORs for MetS components of individuals consuming >3 servings of nuts per week versus those with infrequent nut consumption. As shown, high nut consumers had a lower risk of abdominal obesity (OR = 0.68, 0.60 to 0.79; P-trend<0.001) compared to infrequent consumers of nuts. No associations were shown for high fasting glucose, high blood pressure, high triglycerides, or low HDL-C.

**Table 3:** Multivariable-adjusted odds ratios (95% confidence intervals) for the prevalence of metabolic risk factors by category of nut consumption.

	Nut consumption			
	<1 serving/week(n = 2796)	1–3 servings/week(n = 2125)	>3 servings/week(n = 2289)	P for trend
**Obesity (BMI> = 30 kg/m** 2 **); n = 7210**				
**Unadjusted model**	1 (ref.)	0.76 (0.68–0.86)	0.57 (0.51–0.63)	<0.001
**Model 1**	1 (ref.)	0.78 (0.70–0.88)	0.58 (0.52–0.65)	<0.001
**Model 2**	1 (ref.)	0.80 (0.72–0.90)	0.61 (0.54–0.68)	<0.001
**Model 3**	1 (ref.)	0.80 (0.71–0.90)	0.61 (0.54–0.68)	<0.001
**Diabetes mellitus; n = 7210**				
**Unadjusted model**	1 (ref.)	0.86 (0.77–0.96)	0.81 (0.73–0.91)	0.001
**Model 1**	1 (ref.)	0.85 (0.76–0.96)	0.78 (0.69–0.87)	<0.001
**Model 2**	1 (ref.)	0.86 (0.76–0.96)	0.77 (0.69–0.86)	<0.001
**Model 3**	1 (ref.)	0.91 (0.81–1.02)	0.87 (0.78–0.99)	0.043
**Hypertension; n = 7209**				
**Unadjusted model**	1 (ref.)	0.86 (0.74–1.00)	0.89 (0.77–1.03)	0.251
**Model 1**	1 (ref.)	0.93 (0.80–1.08)	1.02 (0.88–1.19)	0.580
**Model 2**	1 (ref.)	0.91 (0.78–1.06)	1.00 (0.86–1.16)	0.764
**Model 3**	1 (ref.)	0.91 (0.78–1.07)	1.01 (0.87–1.19)	0.602
**Atherogenic dislypidemia** 1 **; n = 6602**				
**Unadjusted model**	1 (ref.)	0.81 (0.68–0.96)	0.74 (0.62–0.88)	0.002
**Model 1**	1 (ref.)	0.84 (0.71–1.00)	0.81 (0.68–0.96)	0.032
**Model 2**	1 (ref.)	0.85 (0.71–1.01)	0.84 (0.70–0.99)	0.083
**Model 3**	1 (ref.)	0.88 (0.74–1.05)	0.89 (0.74–1.07)	0.327
**Hypercholesterolemia (LDL-C >130 mg/dl); n = 6587**				
**Unadjusted model**	1 (ref.)	1.07 (0.95–1.20)	1.05 (0.94–1.18)	0.527
**Model 1**	1 (ref.)	1.07 (0.95–1.20)	1.06 (0.941.20)	0.396
**Model 2**	1 (ref.)	1.07 (0.95–1.20)	1.06 (0.94–1.20)	0.418
**Model 3**	1 (ref.)	1.05 (0.93–1.18)	1.02 (0.90–1.16)	0.857
**Metabolic syndrome** 2 **; (n = 6409)**				
**Unadjusted model**	1 (ref.)	0.79 (0.69–0.89)	0.63 (0.56–0.72)	<0.001
**Model 1**	1 (ref.)	0.81 (0.72–0.93)	0.65 (0.58–0.74)	<0.001
**Model 2**	1 (ref.)	0.84 (0.74–0.95)	0.68 (0.60–0.78)	<0.001
**Model 3**	1 (ref.)	0.87 (0.76–0.99)	0.74 (0.65–0.85)	<0.001

Abbreviations: LDL-C: low-density lipoproteins cholesterol: BMI: body mass index (kg/m^2^).

1 Defined as serum triglycerides ≥150 mg/dL associated with HDL-cholesterol <40 mg/dL in men or <50 mg/dL in women.

2 Metabolic syndrome and obesity were not adjusted by BMI.

Multiple logistic regression was used to assess the association between frequency of nut intake and cardiovascular risk factors.

Multiple logistic regression taking into an account the median of each category of nut consumption was used to generate the P for linear trend.

Model 1 was adjusted for: age (years), sex, geographic recruitment area and BMI (kg/m^2^).

Model 2 was additionally adjusted for smoking status (never, former or current smoker), leisure time physical activity (MET-min/day) and education level (primary or illiterate, secondary and university).

Model 3 was additionally adjusted for energy intake (kcal/day) and adherence to the Mediterranean diet (13-point score). In case of hypercholesterolemia this model was additionally adjusted by treatment with statins.

Extremes of total energy intake were excluded.

**Table 4:** Odds ratios (ORs) and 95% confidence intervals (CIs) for components of metabolic syndrome by category of nut consumption.

	Nut consumption			
	<1 serving/week(n = 2796)	1–3 servings/week(n = 2125)	>3 servings/week(n = 2289)	P for trend
**Abdominal obesity; n = 6999**				
**Unadjusted model**	1 (ref.)	0.84 (0.73–0.96)	0.58 (0.51–0.65)	<0.001
**Model 1**	1 (ref.)	0.87 (0.76–0.99)	0.58 (0.51–0.66)	<0.001
**Model 2**	1 (ref.)	0.92 (0.80–1.06)	0.64 (0.56–0.72)	<0.001
**Model 3**	1 (ref.)	0.96 (0.83-1.10)	0.68 (0.60–0.79)	<0.001
**Hypertriglyceridemia; n = 6611**				
**Unadjusted model**	1 (ref.)	0.84 (0.74–0.96)	0.87 (0.76–0.98)	0.067
**Model 1**	1 (ref.)	0.84 (0.74–0.96)	0.90 (0.79–1.02)	0.216
**Model 2**	1 (ref.)	0.86 (0.75–0.97)	0.92 (0.81–1.05)	0.427
**Model 3**	1 (ref.)	0.87 (0.76–0.99)	0.96 (0.84–1.09)	0.862
**Reduced HDL-C; n = 6617**				
**Unadjusted model**	1 (ref.)	0.92 (0.81–1.04)	0.80 (0.70–0.90)	<0.001
**Model 1**	1 (ref.)	0.94 (0.82–1.06)	0.86 (0.75–0.97)	0.018
**Model 2**	1 (ref.)	0.97 (0.85–1.10)	0.90 (0.79–1.03)	0.121
**Model 3**	1 (ref.)	1.00 (0.88–1.14)	0.98 (0.86–1.12)	0.740
**Elevated blood pressure; n = 6585**				
**Unadjusted model**	1 (ref.)	0.92 (0.69–1.24)	1.01 (0.75–1.36)	0.843
**Model 1**	1 (ref.)	1.00 (0.74–1.34)	1.17 (0.87–1.58)	0.259
**Model 2**	1 (ref.)	0.96 (0.71–1.29)	1.13 (0.83–1.53)	0.368
**Model 3**	1 (ref.)	0.96 (0.71–1.29)	1.12 (0.81–1.53)	0.411
**Elevated fasting glucose; n = 6539**				
**Unadjusted model**	1 (ref.)	0.79 (0.69–0.89)	0.84 (0.74–0.95)	0.047
**Model 1**	1 (ref.)	0.80 (0.70–0.91)	0.84 (0.74–0.95)	0.034
**Model 2**	1 (ref.)	0.80 (0.70–0.91)	0.83 (0.73–0.94)	0.024
**Model 3**	1 (ref.)	0.85 (0.74–0.96)	0.95 (0.83–1.08)	0.845

Model 1 was adjusted for: age (years), sex, geographic recruitment area and BMI (kg/m^2^). The abdominal obesity component of the metabolic syndrome was not adjusted by BMI.

Model 2 was additionally adjusted for smoking status (never, former or current smoker), leisure time physical activity (MET-min/day) and education level (primary or illiterate, secondary and university).

Model 3 was additionally adjusted for energy intake (kcal/day) and adherence to the Mediterranean diet (13-point score).

Extremes of total energy intake were excluded.

Supplementary Tables S1 and S2 show the ORs of each of the confounders used in the multivariate regression analyses in Tables 3 and 4 for the same cardiometabolic risk factors.

## Discussion

In this cross-sectional study conducted in elderly subjects at high cardiovascular risk living in a Mediterranean country, a high frequency of nut consumption was inversely associated with the prevalence of obesity, MetS and diabetes, suggesting that nut consumption has protective effects on cardiometabolic risk. The suggested benefit on MetS was ascribable to a reduced frequency of abdominal obesity, but not of high fasting glucose, hypertension or atherogenic dyslipidemia. These findings may explain in part the decreased risk of CVD mortality shown for frequent nut consumers in prospective studies [7].

The observed inverse association between frequency of nut consumption and prevalence of obesity might seem counterintuitive, since nuts are high-fat, energy-dense foods. However, our results concur not only with previous cross-sectional studies, but also with large prospective cohorts with sufficiently long follow-up showing an inverse association between frequency of nut consumption and BMI or risk of obesity [15], [23]–[25]. They are also in accordance with the results of short and medium-term controlled feeding trials showing that nut supplementation of usual diets does not induce weight gain in spite of the expected increase in energy intake, as recently reviewed [7]. Other clinical trials have suggested that nuts may favor weight loss within energy-restricted diets, possibly by increasing compliance, but enhanced satiety, increased thermogenesis [26], incomplete mastication and fat malabsorption, documented as increased fecal fat excretion in several nut studies [27], could also be contributing factors.

Few studies have examined the association between nut consumption and MetS. In our study the frequency of nut consumption was inversely associated with MetS and with the central obesity component of the MetS. Similar findings were recently reported in the NHANES 1999–2004 cohort, wherein increased nut consumption related to a decreased prevalence of selected CVD risk factors and MetS [28]. In a recent study, including 9987 participants of the SUN cohort followed for 6 years, participants who consumed ≥2 servings of nuts per week had a 32% lower risk of developing MetS than those who never/almost never consumed nuts, and the association appeared to be stronger among women [29]. In addition, 1-y results from a subsample of the first 1224 participants of the PREDIMED randomized trial showed that a Mediterranean diet supplemented with nuts decreased MetS prevalence, mainly by reversing abdominal obesity [30]. Now we include all the participants in the trial in this baseline cross-sectional assessment. In the present study we found no association between nut consumption and the hyperglycemia component of the MetS. An earlier report of the PREDIMED trial with 772 participants and a 3-month follow-up [31] showed that the MedDiet enriched with nuts was associated with improved fasting glucose concentrations. Another cross-sectional assessment in the first 3204 participants supported an inverse association between adherence to the MedDiet and metabolic risk factors (diabetes, obesity and hypertension) [32]. This is the reason why we controlled for overall adherence to the MedDiet using the 13-point score. However the effects of nut consumption on insulin resistance and glycemic control are controversial, especially in individuals with MetS [33]–[36].

In our study subjects who consumed >3 servings of nuts/wk had a 22% lower prevalence of a diagnosis of diabetes than those consuming <1 serving/wk. As reviewed [16], prior epidemiological evidence on the effects of nuts on diabetes risk was inconclusive. Four large prospective studies have evaluated the association between the frequency of nut consumption and the risk of diabetes, the Nurses’ Health Study [37], the Iowa Women’s Health Study [38], the Shanghai Women’s Health Study [39], all carried out in women, and the Physicians’ Health Study, conducted in a cohort of men [40]. After adjustment for confounders, nut consumption was inversely associated with diabetes risk in two studies [37], [39], while no association was found in the other two studies [38], [40]. Several factors may influence the effect of nuts on the pathophysiological process of diabetes. Nuts are rich in unsaturated fatty acids, fiber, magnesium, and other antioxidant and phytochemical constituents with potential beneficial effects on insulin sensitivity and inflammation. In fact, several clinical trials suggest that nuts can modulate oxidative stress [19], inflammation [20], endothelial function [17], and insulin resistance [16], [41]. Recently, nut supplementation has been demonstrated to ameliorate glycemic control in patients with diabetes [42]. Still, reverse causation due to a reduced consumption of fat-rich nuts by diabetic patents on advice of their caregivers is an alternate explanation for the observed inverse association between nut consumption and prevalence of diabetes.

We found no association of nut consumption with clinical hypertension or high blood pressure as MetS component. Prior studies showed conflicting results. Only two prospective studies have evaluated the association between nut consumption and incident hypertension [40], [43]. In a prospective cohort from the Physicians’ Health Study I, Djoussé et al. [40] reported a lower incidence of hypertension in usual consumers of nuts compared to non consumers. However, this association was mainly observed among lean subjects (BMI <25 kg/m^2^) and not in overweight or obese individuals. These results must be taken with caution, however, because salt intake and changes in weight, two major factors that influence the risk of hypertension, were not accounted for in this study. The second study, which involved Spanish university graduates followed for a median of 4.3 years in the SUN cohort [43], found no association between nut consumption and incidence of hypertension after adjusting for several confounders, including exposure to salt and weight changes during follow-up. Also, as recently reviewed [17], few clinical trials have evaluated the effect of nut consumption on blood pressure. Most studies have found either a beneficial effect or no effect, but it must be noted that ambulatory blood pressure monitoring, the best standard for blood pressure measurements, was used in none of these studies. The lack of association between the frequency of nut consumption and hypertension in our study could be explained by the fact that more than 80% of study subjects had a diagnosis of clinical hypertension and more than 90% had high blood pressure as a MetS component.

We observed no association between nut consumption and triglycerides or HDL-C, the lipid components of MetS, or with the simultaneous occurrence of high triglycerides and low HDL-C (atherogenic dyslipidemia). The NHANES 1999–2004 study [28] found that consumers of nuts and peanuts had a 20% lower risk of low HDL-C, while the 3-mo report of the first 772 participants in the PREDIMED trial [31] showed reduced triglycerides and increased HDL-C with the nut-enriched MedDiet. However, a recent pooled analysis of clinical studies showed that nut consumption had no effect on HDL-C, although it reduced triglycerides when they were elevated at baseline [18]. This report also showed that nut intake induces a consistent, dose-related reduction of total cholesterol and LDL-C [18], which is counter to the lack of association between nut consumption and LDL-C in our study. However, this might be due to the high prevalence of obesity, a condition which appears to blunt the hypocholesterolemic effect of nuts [18], [33].

The present study has some limitations. First, the cross-sectional design limits the potential to discern causative relationships. Second, the results cannot be extrapolated to the general population because the analysis was conducted in an older Mediterranean population at high cardiovascular risk. Third, we adjusted the regression models for physical activity, tobacco and adherence to the MedDiet in order to control for a wide array of confounding factors, including lifestyle factors and potential dietary confounders. However, we acknowledge that we cannot discount residual confounding, namely that factors unaccounted for in the questionnaires that imply a healthier lifestyle could mediate the inverse association between nut consumption and cardiometabolic risk factors. Our study also has important strengths**.** Only two studies have analysed the association between nut consumption and MetS or atherogenic dyslipidemia. The present epidemiologic study is the first showing an association between the frequency of nut consumption and MetS in individuals at high cardiovascular risk. Another strength is the large size and wide geographical origin within Spain of the population studied.

In conclusion, in a Mediterranean population at high risk for CVD, the frequency of nut consumption was inversely associated with obesity, MetS, and diabetes prevalence after adjusting for potential confounding factors. Further research is needed to identify the mechanisms by which nuts improve cardiometabolic risk. It also remains to be explored whether residual confounding related to a healthier lifestyle of nut eaters might explain in part the benefits observed in this study.

## Supporting Information

Table S1 **Multivariable-adjusted odds ratios (95% confidence intervals) for the prevalence of metabolic risk factors by category of nut consumption.**
(DOC)

Table S2 **Multivariable-adjusted odds ratios (95% confidence intervals) for the risk of components of metabolic syndrome by category of nut consumption.**
(DOC)
