# Supplementary material for: Cross-Sectional Assessment of Nut Consumption and Obesity, Metabolic Syndrome and Other Cardiometabolic Risk Factors: The PREDIMED Study
Source: PLoS One. 2013 Feb 27;8(2):e57367. doi: 10.1371/journal.pone.0057367 (PMC3583833; doi:10.1371/journal.pone.0057367)
Supplement: Table S1 — Multivariable-adjusted odds ratios (95% confidence intervals) for the prevalence of metabolic risk factors by category of nut consumption. (DOC) [file pone.0057367.s001.doc]

**SUPPLEMENTARY FILE**

**Table S1. Multivariable-adjusted odds ratios (95% confidence intervals) for the prevalence of metabolic risk factors by category of nut consumption.**

| **Obesity (BMI ≥ 30kg/m^2^)** | | | |
| --- | --- | --- | --- |
|  | **Odds ratio** | **95% CI** | |
| **Model unadjusted** |  |  |  |
| **< 1 serving/week** | 1 (ref.) |  |  |
| **1-3 servings/week** | 0.76 | 0.68 | 0.86 |
| **>3 servings/week** | 0.57 | 0.51 | 0.63 |
| **Model 1** |  |  |  |
| **< 1 serving/week** | 1 (ref.) |  |  |
| **1-3 servings/week** | 0.78 | 0.70 | 0.88 |
| **>3 servings/week** | 0.58 | 0.52 | 0.65 |
| **Node** | 0.99 | 0.98 | 0.65 |
| **Age (years)** | 0.99 | 0.98 | 1.00 |
| **Sex (women)** | 1.58 | 1.43 | 1.73 |
| **Model 2** |  |  |  |
| **< 1 serving/week** | 1 (ref.) |  |  |
| **1-3 servings/week** | 0.80 | 0.72 | 0.90 |
| **>3 servings/week** | 0.61 | 0.54 | 0.68 |
| **Node** | 0.99 | 0.98 | 1.00 |
| **Age (years)** | 0.98 | 0.97 | 0.99 |
| **Sex (women)** | 1.20 | 1.06 | 1.36 |
| **Smoking status (never)** | 1 (ref.) |  |  |
| **Smoking status (current smoker)** | 0.68 | 0.58 | 0.80 |
| **Smoking status (former)** | 0.94 | 0.82 | 1.08 |
| **Physical activity (x 100 MET-min/day)** | 0.91 | 0.89 | 0.93 |
| **Level education (primary or illiterate)** | 1 (ref.) |  |  |
| **Level education (secondary)** | 0.74 | 0.64 | 0.85 |
| **Level education (university)** | 0.66 | 0.54 | 0.80 |
| **Model 3** |  |  |  |
| **< 1 serving/week** | 1 (ref.) |  |  |
| **1-3 servings/week** | 0.80 | 0.71 | 0.90 |
| **>3 servings/week** | 0.61 | 0.54 | 0.68 |
| **Node** | 0.99 | 0.98 | 1.00 |
| **Age (years)** | 0.98 | 0.98 | 0.99 |
| **Sex (women)** | 1.22 | 1.08 | 1.39 |
| **Smoking status (never)** |  |  |  |
| **Smoking status (current smoker)** | 0.67 | 0.57 | 0.79 |
| **Smoking status (former)** | 0.94 | 0.82 | 1.08 |
| **Physical activity (x 100 MET-min/day)** | 0.91 | 0.89 | 0.93 |
| **Level education (primary or illiterate)** |  |  |  |
| **Level education (secondary)** | 0.74 | 0.64 | 0.85 |
| **Level education (university)** | 0.66 | 0.55 | 0.81 |
| **13-point Score** | 0.95 | 0.92 | 0.98 |
| **Total energy intake (x 1000 Kcal/day)** | 1.09 | 0.99 | 1.19 |

| **Diabetes** | | | |
| --- | --- | --- | --- |
|  | **Odds ratio** | **95% CI** | |
| **Model unadjusted** |  |  |  |
| **< 1 serving/week** | 1 (ref.) |  |  |
| **1-3 servings/week** | 0.86 | 0.77 | 0.96 |
| **>3 servings/week** | 0.81 | 0.73 | 0.91 |
| **Model 1** |  |  |  |
| **< 1 serving/week** | 1 (ref.) |  |  |
| **1-3 servings/week** | 0.85 | 0.76 | 0.96 |
| **>3 servings/week** | 0.78 | 0.69 | 0.87 |
| **Node** | 0.98 | 0.97 | 0.99 |
| **Age** | 1.03 | 1.02 | 1.03 |
| **Sex (women)** | 0.64 | 0.58 | 0.70 |
| **BMI (kg/m^2^)** | 1.00 | 0.98 | 1.01 |
| **Model 2** |  |  |  |
| **< 1 serving/week** | 1 (ref.) |  |  |
| **1-3 servings/week** | 0.86 | 0.76 | 0.96 |
| **>3 servings/week** | 0.77 | 0.69 | 0.86 |
| **Node** | 0.97 | 0.96 | 0.99 |
| **Age** | 1.02 | 1.01 | 1.03 |
| **Sex (women)** | 0.53 | 0.47 | 0.60 |
| **BMI (kg/m^2^)** | 0.99 | 0.98 | 1.00 |
| **Smoking status (never)** | 1 (ref.) |  |  |
| **Smoking status (current smoker)** | 0.59 | 0.50 | 0.69 |
| **Smoking status (former)** | 0.91 | 0.79 | 1.05 |
| **Physical activity (x 100 MET-min/day)** | 1.00 | 0.98 | 1.03 |
| **Level education (primary or illiterate)** | 1 (ref.) |  |  |
| **Level education (secondary)** | 0.77 | 0.67 | 0.88 |
| **Level education (university)** | 0.65 | 0.54 | 0.79 |
| **Model 3** |  |  |  |
| **< 1 serving/week** | 1 (ref.) |  |  |
| **1-3 servings/week** | 0.91 | 0.81 | 1.02 |
| **>3 servings/week** | 0.87 | 0.78 | 0.99 |
| **Node** | 0.97 | 0.96 | 0.98 |
| **Age** | 1.02 | 1.01 | 1.02 |
| **Sex (women)** | 0.48 | 0.42 | 0.55 |
| **BMI (kg/m^2^)** | 0.99 | 0.98 | 1.00 |
| **Smoking status (never)** | 1 (ref.) |  |  |
| **Smoking status (current smoker)** | 0.60 | 0.51 | 0.71 |
| **Smoking status (former)** | 0.91 | 0.79 | 1.04 |
| **Physical activity (x 100 MET-min/day)** | 1.01 | 0.99 | 1.03 |
| **Level education (primary or illiterate)** | 1 (ref.) |  |  |
| **Level education (secondary)** | 0.78 | 0.68 | 0.90 |
| **Level education (university)** | 0.67 | 0.55 | 0.81 |
| **13-point Score** | 0.95 | 0.92 | 0.97 |
| **Total energy intake (x 1000 Kcal/day)** | 0.71 | 0.64 | 0.78 |

| **Hypertension** | | | |
| --- | --- | --- | --- |
|  | **Odds ratio** | **95% CI** | |
| **Model unadjusted** |  |  |  |
| **< 1 serving/week** | 1 (ref.) |  |  |
| **1-3 servings/week** | 0.86 | 0.74 | 1.00 |
| **>3 servings/week** | 0.89 | 0.77 | 1.03 |
| **Model 1** |  |  |  |
| **< 1 serving/week** | 1 (ref.) |  |  |
| **1-3 servings/week** | 0.93 | 0.80 | 1.08 |
| **>3 servings/week** | 1.02 | 0.88 | 1.19 |
| **Node** | 1.01 | 1.00 | 1.03 |
| **Age** | 1.03 | 1.02 | 1.04 |
| **Sex (women)** | 1.57 | 1.38 | 1.78 |
| **BMI (kg/m^2^)** | 1.10 | 1.08 | 1.12 |
| **Model 2** |  |  |  |
| **< 1 serving/week** | 1 (ref.) |  |  |
| **1-3 servings/week** | 0.91 | 0.78 | 1.06 |
| **>3 servings/week** | 1.00 | 0.86 | 1.16 |
| **Node** | 1.01 | 1.00 | 1.03 |
| **Age** | 1.03 | 1.02 | 1.04 |
| **Sex (women)** | 1.35 | 1.15 | 1.59 |
| **BMI (kg/m^2^)** | 1.10 | 1.08 | 1.12 |
| **Smoking status (never)** | 1 (ref.) |  |  |
| **Smoking status (current smoker)** | 0.62 | 0.51 | 0.76 |
| **Smoking status (former)** | 0.81 | 0.68 | 0.96 |
| **Physical activity (x 100 MET-min/day)** | 0.99 | 0.96 | 1.01 |
| **Level education (primary or illiterate)** | 1 (ref.) |  |  |
| **Level education (secondary)** | 1.28 | 1.07 | 1.53 |
| **Level education (university)** | 1.35 | 1.06 | 1.72 |
| **Model 3** |  |  |  |
| **< 1 serving/week** | 1 (ref.) |  |  |
| **1-3 servings/week** | 0.91 | 0.78 | 1.07 |
| **>3 servings/week** | 1.01 | 0.87 | 1.19 |
| **Node** | 1.01 | 1.00 | 1.03 |
| **Age** | 1.03 | 1.02 | 1.04 |
| **Sex (women)** | 1.34 | 1.14 | 1.58 |
| **BMI (kg/m^2^)** | 1.10 | 1.08 | 1.12 |
| **Smoking status (never)** | 1 (ref.) |  |  |
| **Smoking status (current smoker)** | 0.62 | 0.51 | 0.76 |
| **Smoking status (former)** | 0.80 | 0.68 | 0.96 |
| **Physical activity (x 100 MET-min/day)** | 0.99 | 0.96 | 1.01 |
| **Level education (primary or illiterate)** | 1 (ref.) |  |  |
| **Level education (secondary)** | 1.29 | 1.08 | 1.54 |
| **Level education (university)** | 1.36 | 1.06 | 1.73 |
| **13-point Score** | 0.98 | 0.95 | 1.02 |
| **Total energy intake (x 1000 Kcal/day)** | 0.97 | 0.86 | 1.10 |

| **Atherogenic dislypidemia** | | | |
| --- | --- | --- | --- |
|  | **Odds ratio** | **95% CI** | |
| **Model unadjusted** |  |  |  |
| **< 1 serving/week** | 1 (ref.) |  |  |
| **1-3 servings/week** | 0.81 | 0.68 | 0.96 |
| **>3 servings/week** | 0.74 | 0.62 | 0.88 |
| **Model 1** |  |  |  |
| **< 1 serving/week** | 1 (ref.) |  |  |
| **1-3 servings/week** | 0.84 | 0.71 | 1.00 |
| **>3 servings/week** | 0.81 | 0.68 | 0.96 |
| **Node** | 1.02 | 1.00 | 1.04 |
| **Age** | 1.00 | 0.99 | 1.01 |
| **BMI (kg/m^2^)** | 1.08 | 1.06 | 1.10 |
| **Model 2** |  |  |  |
| **< 1 serving/week** | 1 (ref.) |  |  |
| **1-3 servings/week** | 0.85 | 0.71 | 1.01 |
| **>3 servings/week** | 0.84 | 0.70 | 0.99 |
| **Node** | 1.02 | 1.00 | 1.04 |
| **Age** | 1.00 | 0.99 | 1.01 |
| **BMI (kg/m^2^)** | 1.08 | 1.06 | 1.10 |
| **Smoking status (never)** | 1 (ref.) |  |  |
| **Smoking status (current smoker)** | 1.03 | 0.83 | 1.29 |
| **Smoking status (former)** | 0.77 | 0.63 | 0.93 |
| **Physical activity (x 100 MET-min/day)** | 0.95 | 0.91 | 0.98 |
| **Level education (primary or illiterate)** | 1 (ref.) |  |  |
| **Level education (secondary)** | 0.99 | 0.80 | 1.23 |
| **Level education (university)** | 1.25 | 0.94 | 1.65 |
| **Model 3** |  |  |  |
| **< 1 serving/week** | 1 (ref.) |  |  |
| **1-3 servings/week** | 0.88 | 0.74 | 1.05 |
| **>3 servings/week** | 0.89 | 0.74 | 1.07 |
| **Node** | 1.02 | 1.00 | 1.03 |
| **Age** | 1.00 | 0.99 | 1.01 |
| **BMI (kg/m^2^)** | 1.08 | 1.06 | 1.10 |
| **Smoking status (never)** | 1 (ref.) |  |  |
| **Smoking status (current smoker)** | 1.05 | 0.84 | 1.31 |
| **Smoking status (former)** | 0.78 | 0.64 | 0.94 |
| **Physical activity (x 100 MET-min/day)** | 0.95 | 0.92 | 0.99 |
| **Level education (primary or illiterate)** | 1 (ref.) |  |  |
| **Level education (secondary)** | 1.00 | 0.81 | 1.24 |
| **Level education (university)** | 1.27 | 0.96 | 1.68 |
| **13-point Score** | 0.94 | 0.90 | 0.98 |
| **Total energy intake (x 1000 Kcal/day)** | 0.90 | 0.78 | 1.03 |

| **Hypercholesterolemia** | | | |
| --- | --- | --- | --- |
|  | **Odds ratio** | **95% CI** | |
| **Unadjusted model** |  |  |  |
| **< 1 serving/week** | 1 (ref.) |  |  |
| **1-3 servings/week** | 1.07 | 0.95 | 1.20 |
| **>3 servings/week** | 1.05 | 0.94 | 1.18 |
| **Model 1** |  |  |  |
| **< 1 serving/week** | 1 (ref.) |  |  |
| **1-3 servings/week** | 1.07 | 0.95 | 1.20 |
| **>3 servings/week** | 1.06 | 0.95 | 1.20 |
| **Node** | 0.98 | 0.97 | 0.99 |
| **Age** | 0.98 | 0.97 | 0.98 |
| **Sex (women)** | 1.44 | 1.30 | 1.59 |
| **BMI** | 0.99 | 0.98 | 1.00 |
| **Model 2** |  |  |  |
| **< 1 serving/week** | 1 (ref.) |  |  |
| **1-3 servings/week** | 1.07 | 0.95 | 1.20 |
| **>3 servings/week** | 1.06 | 0.94 | 1.20 |
| **Node** | 0.98 | 0.97 | 0.99 |
| **Age** | 0.98 | 0.97 | 0.99 |
| **Sex (women)** | 1.47 | 1.29 | 1.68 |
| **BMI (kg/m^2^)** | 0.99 | 0.98 | 1.00 |
| **Smoking status (never)** | 1 (ref.) |  |  |
| **Smoking status (current smoker)** | 1.06 | 0.90 | 1.25 |
| **Smoking status (former)** | 0.99 | 0.86 | 1.14 |
| **Physical activity (x 100 MET-min/day)** | 1.01 | 0.99 | 1.04 |
| **Level education (primary or illiterate)** | 1 (ref.) |  |  |
| **Level education (secondary)** | 0.97 | 0.85 | 1.12 |
| **Level education (university)** | 1.02 | 0.84 | 1.23 |
| **Model 3** |  |  |  |
| **< 1 serving/week** | 1 (ref.) |  |  |
| **1-3 servings/week** | 1.05 | 0.93 | 1.18 |
| **>3 servings/week** | 1.02 | 0.90 | 1.16 |
| **Node** | 0.98 | 0.97 | 1.00 |
| **Age** | 0.98 | 0.97 | 0.98 |
| **Sex (women)** | 1.63 | 1.42 | 1.87 |
| **BMI (kg/m^2^)** | 0.98 | 0.97 | 1.00 |
| **Smoking status (never)** | 1 (ref.) |  |  |
| **Smoking status (current smoker)** | 1.02 | 0.86 | 1.21 |
| **Smoking status (former)** | 1.00 | 0.86 | 1.16 |
| **Physical activity (x 100 MET-min/day)** | 1.02 | 0.99 | 1.04 |
| **Level education (primary or illiterate)** | 1 (ref.) |  |  |
| **Level education (secondary)** | 0.96 | 0.83 | 1.10 |
| **Level education (university)** | 1.01 | 0.83 | 1.23 |
| **13-point Score** | 1.02 | 0.99 | 1.05 |
| **Total energy intake (x 1000 Kcal/day)** | 1.07 | 0.97 | 1.19 |
| **Treatment with statins** | 0.42 | 0.38 | 0.46 |

| **Metabolic Syndrome^2^** | | | |
| --- | --- | --- | --- |
|  | **Odds ratio** | **95% CI** | |
| **Unadjusted model** |  |  |  |
| **< 1 serving/week** | 1 (ref.) |  |  |
| **1-3 servings/week** | 0.79 | 0.69 | 0.89 |
| **>3 servings/week** | 0.63 | 0.56 | 0.72 |
| **Model 1** |  |  |  |
| **< 1 serving/week** | 1 (ref.) |  |  |
| **1-3 servings/week** | 0.81 | 0.72 | 0.93 |
| **>3 servings/week** | 0.65 | 0.58 | 0.74 |
| **Node** | 0.97 | 0.96 | 0.99 |
| **Age** | 1.01 | 1.00 | 1.02 |
| **Sex (women)** | 1.49 | 1.34 | 1.66 |
| **Model 2** |  |  |  |
| **< 1 serving/week** | 1 (ref.) |  |  |
| **1-3 servings/week** | 0.84 | 0.74 | 0.95 |
| **>3 servings/week** | 0.68 | 0.60 | 0.78 |
| **Node** | 0.98 | 0.96 | 0.99 |
| **Age** | 1.00 | 0.99 | 1.01 |
| **Sex (women)** | 1.31 | 1.14 | 1.50 |
| **Smoking status (never)** | 1 (ref.) |  |  |
| **Smoking status (current smoker)** | 0.97 | 0.81 | 1.15 |
| **Smoking status (former)** | 1.07 | 0.92 | 1.25 |
| **Physical activity (x 100 MET-min/day)** | 0.93 | 0.91 | 0.95 |
| **Level education (primary or illiterate)** | 1 (ref.) |  |  |
| **Level education (secondary)** | 0.76 | 0.66 | 0.88 |
| **Level education (university)** | 0.69 | 0.57 | 0.84 |
| **Model 3** |  |  |  |
| **< 1 serving/week** | 1 (ref.) |  |  |
| **1-3 servings/week** | 0.87 | 0.76 | 0.99 |
| **>3 servings/week** | 0.74 | 0.65 | 0.85 |
| **Node** | 0.97 | 0.96 | 0.98 |
| **Age** | 1.00 | 0.99 | 1.01 |
| **Sex (women)** | 1.26 | 1.09 | 1.45 |
| **Smoking status (never)** | 1 (ref.) |  |  |
| **Smoking status (current smoker)** | 0.97 | 0.81 | 1.15 |
| **Smoking status (former)** | 1.07 | 0.92 | 1.25 |
| **Physical activity (x 100 MET-min/day)** | 0.93 | 0.91 | 0.96 |
| **Level education (primary or illiterate)** | 1 (ref.) |  |  |
| **Level education (secondary)** | 0.76 | 0.66 | 0.88 |
| **Level education (university)** | 0.71 | 0.58 | 0.86 |
| **13-point Score** | 0.92 | 0.90 | 0.95 |
| **Total energy intake (x 1000 Kcal/day)** | 0.86 | 0.78 | 0.95 |

Abbreviations: LDL-C: low-density lipoproteins cholesterol: BMI: body mass index (kg/m^2^).

^1^Defined as serum triglycerides ≥150 mg/dL associated with HDL-cholesterol <40 mg/dL in men or <50 mg/dL in women.

^2^Metabolic syndrome was not adjusted by BMI.

Multiple logistic regression was used to assess the association between frequency of nut intake and cardiovascular risk factors.

Multiple logistic regression taking into an account the median of each category of nut consumption was used to generate the P for linear trend.

Model 1 was adjusted for: age (years), sex, geographic recruitment area and BMI (kg/m^2^).

Model 2 was additionally adjusted for smoking status (never, former or current smoker), leisure time physical activity (x 100 MET-min/day) and level education (primary or illiterate, secondary and university).

Model 3 was additionally adjusted for energy intake (x 1000 kcal/day) and adherence to the Mediterranean diet (13-point score). Hypercholesterolemia was additionally adjusted by statin treatment.

Extremes of total energy intake were excluded.
