# Supplementary material for: Cross-Sectional Assessment of Nut Consumption and Obesity, Metabolic Syndrome and Other Cardiometabolic Risk Factors: The PREDIMED Study
Source: PLoS One. 2013 Feb 27;8(2):e57367. doi: 10.1371/journal.pone.0057367 (PMC3583833; doi:10.1371/journal.pone.0057367)
Supplement: Table S2 — Multivariable-adjusted odds ratios (95% confidence intervals) for the risk of components of metabolic syndrome by category of nut consumption. (DOC) [file pone.0057367.s002.doc]

**SUPPLEMENTARY FILE**

**Table S2. Multivariable-adjusted odds ratios (95% confidence intervals) for the risk of components of metabolic syndrome by category of nut consumption.**

| **Abdominal obesity** | | | |
| --- | --- | --- | --- |
|  | **Odds ratio** | **95% CI** | |
| **Unadjusted model** |  |  |  |
| **< 1 serving/week** | 1 (ref.) |  |  |
| **1-3 servings/week** | 0.84 | 0.73 | 0.96 |
| **>3 servings/week** | 0.58 | 0.51 | 0.65 |
| **Model 1** |  |  |  |
| **< 1 serving/week** | 1 (ref.) |  |  |
| **1-3 servings/week** | 0.87 | 0.76 | 0.99 |
| **>3 servings/week** | 0.58 | 0.51 | 0.66 |
| **Node** | 0.98 | 0.96 | 0.99 |
| **Age** | 1.03 | 1.02 | 1.04 |
| **Model 2** |  |  |  |
| **< 1 serving/week** | 1 (ref.) |  |  |
| **1-3 servings/week** | 0.92 | 0.80 | 1.06 |
| **>3 servings/week** | 0.64 | 0.56 | 0.72 |
| **Node** | 0.98 | 0.96 | 0.99 |
| **Age** | 1.02 | 1.01 | 1.03 |
| **Smoking status (never)** | 1 (ref.) |  |  |
| **Smoking status (current smoker)** | 0.46 | 0.40 | 0.54 |
| **Smoking status (former)** | 0.54 | 0.48 | 0.62 |
| **Physical activity (x 100 MET-min/day)** | 0.88 | 0.86 | 0.90 |
| **Level education (primary or illiterate)** | 1 (ref.) |  |  |
| **Level education (secondary)** | 0.72 | 0.62 | 0.84 |
| **Level education (university)** | 0.66 | 0.54 | 0.81 |
| **Model 3** |  |  |  |
| **< 1 serving/week** | 1 (ref.) |  |  |
| **1-3 servings/week** | 0.96 | 0.83 | 1.10 |
| **>3 servings/week** | 0.68 | 0.60 | 0.79 |
| **Node** | 0.97 | 0.96 | 0.99 |
| **Age** | 1.02 | 1.01 | 1.03 |
| **Smoking status (never)** | 1 (ref.) |  |  |
| **Smoking status (current smoker)** | 0.47 | 0.40 | 0.55 |
| **Smoking status (former)** | 0.55 | 0.49 | 0.63 |
| **Physical activity (x 100 MET-min/day)** | 0.88 | 0.86 | 0.90 |
| **Level education (primary or illiterate)** | 1 (ref.) |  |  |
| **Level education (secondary)** | 0.73 | 0.63 | 0.85 |
| **Level education (university)** | 0.68 | 0.55 | 0.83 |
| **13-point Score** | 0.93 | 0.90 | 0.96 |
| **Total energy intake (x 1000 Kcal/day)** | 0.88 | 0.79 | 0.98 |

| **Hypertriglyceridemia** | | | |
| --- | --- | --- | --- |
|  | **Odds ratio** | **95% CI** | |
| **Unadjusted model** |  |  |  |
| **< 1 serving/week** | 1 (ref.) |  |  |
| **1-3 servings/week** | 0.84 | 0.74 | 0.96 |
| **>3 servings/week** | 0.87 | 0.76 | 0.98 |
| **Model 1** |  |  |  |
| **< 1 serving/week** | 1 (ref.) |  |  |
| **1-3 servings/week** | 0.84 | 0.74 | 0.96 |
| **>3 servings/week** | 0.90 | 0.79 | 1.02 |
| **Node** | 1.00 | 0.99 | 1.02 |
| **Age** | 0.99 | 0.98 | 1.00 |
| **Sex (women)** | 0.76 | 0.69 | 0.85 |
| **BMI (kg/m^2^)** | 1.06 | 1.05 | 1.08 |
| **Model 2** |  |  |  |
| **< 1 serving/week** | 1 (ref.) |  |  |
| **1-3 servings/week** | 0.86 | 0.75 | 0.97 |
| **>3 servings/week** | 0.92 | 0.81 | 1.05 |
| **Node** | 1.00 | 0.99 | 1.02 |
| **Age** | 0.99 | 0.98 | 1.00 |
| **Sex (women)** | 0.80 | 0.69 | 0.92 |
| **BMI (kg/m^2^)** | 1.06 | 1.05 | 1.08 |
| **Smoking status (never)** | 1 (ref) |  |  |
| **Smoking status (current smoker)** | 1.35 | 1.14 | 1.61 |
| **Smoking status (former)** | 1.10 | 0.94 | 1.28 |
| **Physical activity (x 100 MET-min/day)** | 0.96 | 0.93 | 0.98 |
| **Level education (primary or illiterate)** | 1 (ref) |  |  |
| **Level education (secondary)** | 0.98 | 0.84 | 1.13 |
| **Level education (university)** | 1.11 | 0.91 | 1.37 |
| **Model 3** |  |  |  |
| **< 1 serving/week** | 1 (ref.) |  |  |
| **1-3 servings/week** | 0.87 | 0.76 | 0.99 |
| **>3 servings/week** | 0.96 | 0.84 | 1.09 |
| **Node** | 1.00 | 0.99 | 1.01 |
| **Age** | 0.99 | 0.98 | 1.00 |
| **Sex (women)** | 0.79 | 0.68 | 0.91 |
| **BMI (kg/m^2^)** | 1.06 | 1.04 | 1.07 |
| **Smoking status (never)** | 1 (ref) |  |  |
| **Smoking status (current smoker)** | 1.35 | 1.13 | 1.60 |
| **Smoking status (former)** | 1.10 | 0.94 | 1.28 |
| **Physical activity (x 100 MET-min/day)** | 0.96 | 0.94 | 0.98 |
| **Level education (primary or illiterate)** | 1 (ref) |  |  |
| **Level education (secondary)** | 0.98 | 0.84 | 1.14 |
| **Level education (university)** | 1.13 | 0.92 | 1.38 |
| **13-point Score** | 0.95 | 0.92 | 0.98 |
| **Total energy intake (x 1000 Kcal/day)** | 0.96 | 0.86 | 1.06 |

| **Reduced HDL-C** | | | |
| --- | --- | --- | --- |
|  | **Odds ratio** | **95% CI** | |
| **Unadjusted model** |  |  |  |
| **< 1 serving/week** | 1 (ref.) |  |  |
| **1-3 servings/week** | 0.92 | 0.81 | 1.04 |
| **>3 servings/week** | 0.80 | 0.70 | 0.90 |
| **Model 1** |  |  |  |
| **< 1 serving/week** | 1 (ref.) |  |  |
| **1-3 servings/week** | 0.94 | 0.82 | 1.06 |
| **>3 servings/week** | 0.86 | 0.75 | 0.97 |
| **Node** | 1.03 | 1.01 | 1.04 |
| **Age** | 0.99 | 0.98 | 1.00 |
| **BMI (kg/m^2^)** | 1.07 | 1.06 | 1.09 |
| **Model 2** |  |  |  |
| **< 1 serving/week** | 1 (ref.) |  |  |
| **1-3 servings/week** | 0.97 | 0.85 | 1.10 |
| **>3 servings/week** | 0.90 | 0.79 | 1.03 |
| **Node** | 1.03 | 1.01 | 1.04 |
| **Age** | 0.99 | 0.98 | 1.00 |
| **BMI (kg/m^2^)** | 1.06 | 1.05 | 1.08 |
| **Smoking status (never)** | 1 (ref.) |  |  |
| **Smoking status (current smoker)** | 1.03 | 0.88 | 1.21 |
| **Smoking status (former)** | 0.76 | 0.67 | 0.87 |
| **Physical activity (x 100 MET-min/day)** | 0.93 | 0.91 | 0.95 |
| **Level education (primary or illiterate)** | 1 (ref.) |  |  |
| **Level education (secondary)** | 0.87 | 0.75 | 1.02 |
| **Level education (university)** | 0.96 | 0.78 | 1.19 |
| **Model 3** |  |  |  |
| **< 1 serving/week** | 1 (ref.) |  |  |
| **1-3 servings/week** | 1.00 | 0.88 | 1.14 |
| **>3 servings/week** | 0.98 | 0.86 | 1.12 |
| **Node** | 1.02 | 1.00 | 1.04 |
| **Age** | 0.99 | 0.98 | 1.00 |
| **BMI (kg/m^2^)** | 1.06 | 1.05 | 1.08 |
| **Smoking status (never)** | 1 (ref.) |  |  |
| **Smoking status (current smoker)** | 1.07 | 0.91 | 1.26 |
| **Smoking status (former)** | 0.79 | 0.69 | 0.90 |
| **Physical activity (x 100 MET-min/day)** | 0.93 | 0.91 | 0.96 |
| **Level education (primary or illiterate)** | 1 (ref.) |  |  |
| **Level education (secondary)** | 0.89 | 0.76 | 1.04 |
| **Level education (university)** | 0.98 | 0.80 | 1.22 |
| **13-point Score** | 0.97 | 0.94 | 1.00 |
| **Total energy intake (x 1000 Kcal/day)** | 0.81 | 0.73 | 0.91 |

| **Elevated blood pressure** | | | |
| --- | --- | --- | --- |
|  | **Odds ratio** | **95% CI** | |
| **Unadjusted model** |  |  |  |
| **< 1 serving/week** | 1 (ref.) |  |  |
| **1-3 servings/week** | 0.92 | 0.69 | 1.24 |
| **>3 servings/week** | 1.01 | 0.75 | 1.36 |
| **Model 1** |  |  |  |
| **< 1 serving/week** | 1 (ref.) |  |  |
| **1-3 servings/week** | 1.00 | 0.74 | 1.34 |
| **>3 servings/week** | 1.17 | 0.87 | 1.58 |
| **Node** | 1.05 | 1.02 | 1.08 |
| **Age** | 1.09 | 1.07 | 1.12 |
| **Sex (women)** | 0.94 | 0.73 | 1.21 |
| **BMI (kg/m^2^)** | 1.17 | 1.13 | 1.21 |
| **Model 2** |  |  |  |
| **< 1 serving/week** | 1 (ref.) |  |  |
| **1-3 servings/week** | 0.96 | 0.71 | 1.29 |
| **>3 servings/week** | 1.13 | 0.83 | 1.53 |
| **Node** | 1.05 | 1.02 | 1.08 |
| **Age** | 1.09 | 1.07 | 1.12 |
| **Sex (women)** | 0.81 | 0.59 | 1.12 |
| **BMI (kg/m^2^)** | 1.17 | 1.12 | 1.21 |
| **Smoking status (never)** | 1 (ref) |  |  |
| **Smoking status (current smoker)** | 0.59 | 0.41 | 0.85 |
| **Smoking status (former)** | 0.76 | 0.53 | 1.08 |
| **Physical activity (x 100 MET-min/day)** | 1.00 | 0.95 | 1.05 |
| **Level education (primary or illiterate)** | 1 (ref) |  |  |
| **Level education (secondary)** | 1.40 | 0.98 | 1.98 |
| **Level education (university)** | 1.89 | 1.11 | 3.23 |
| **Model 3** |  |  |  |
| **< 1 serving/week** | 1 (ref.) |  |  |
| **1-3 servings/week** | 0.96 | 0.71 | 1.29 |
| **>3 servings/week** | 1.12 | 0.81 | 1.53 |
| **Node** | 1.05 | 1.02 | 1.09 |
| **Age** | 1.09 | 1.07 | 1.12 |
| **Sex (women)** | 0.81 | 0.58 | 1.12 |
| **BMI (kg/m^2^)** | 1.17 | 1.13 | 1.21 |
| **Smoking status (never)** | 1 (ref) |  |  |
| **Smoking status (current smoker)** | 0.59 | 0.41 | 0.85 |
| **Smoking status (former)** | 0.76 | 0.53 | 1.08 |
| **Physical activity (x 100 MET-min/day)** | 1.00 | 0.95 | 1.05 |
| **Level education (primary or illiterate)** | 1 (ref) |  |  |
| **Level education (secondary)** | 1.39 | 0.98 | 1.98 |
| **Level education (university)** | 1.88 | 1.10 | 3.21 |
| **13-point Score** | 1.03 | 0.96 | 1.11 |
| **Total energy intake (x 1000 Kcal/day)** | 1.03 | 0.96 | 1.11 |

| **Elevated fasting plasma glucose** | | | |
| --- | --- | --- | --- |
|  | **Odds ratio** | **95% CI** | |
| **Unadjusted model** |  |  |  |
| **< 1 serving/week** | 1 (ref.) |  |  |
| **1-3 servings/week** | 0.79 | 0.69 | 0.89 |
| **>3 servings/week** | 0.84 | 0.74 | 0.95 |
| **Model 1** |  |  |  |
| **< 1 serving/week** | 1 (ref.) |  |  |
| **1-3 servings/week** | 0.80 | 0.70 | 0.91 |
| **>3 servings/week** | 0.84 | 0.74 | 0.95 |
| **Node** | 0.96 | 0.95 | 0.98 |
| **Age** | 1.02 | 1.01 | 1.03 |
| **Sex (women)** | 0.55 | 0.49 | 0.61 |
| **BMI (kg/m^2^)** | 1.04 | 1.03 | 1.06 |
| **Model 2** |  |  |  |
| **< 1 serving/week** | 1 (ref.) |  |  |
| **1-3 servings/week** | 0.80 | 0.70 | 0.91 |
| **>3 servings/week** | 0.83 | 0.73 | 0.94 |
| **Node** | 0.96 | 0.95 | 0.97 |
| **Age** | 1.02 | 1.01 | 1.03 |
| **Sex (women)** | 0.50 | 0.44 | 0.58 |
| **BMI (kg/m^2^)** | 1.04 | 1.02 | 1.05 |
| **Smoking status (never)** | 1 (ref.) |  |  |
| **Smoking status (current smoker)** | 0.72 | 0.60 | 0.86 |
| **Smoking status (former)** | 1.06 | 0.90 | 1.24 |
| **Physical activity (x 100 MET-min/day)** | 1.02 | 0.99 | 1.04 |
| **Level education (primary or illiterate)** | 1 (ref.) |  |  |
| **Level education (secondary)** | 0.77 | 0.66 | 0.89 |
| **Level education (university)** | 0.62 | 0.51 | 0.76 |
| **Model 3** |  |  |  |
| **< 1 serving/week** | 1 (ref.) |  |  |
| **1-3 servings/week** | 0.85 | 0.74 | 0.96 |
| **>3 servings/week** | 0.95 | 0.83 | 1.08 |
| **Node** | 0.95 | 0.94 | 0.97 |
| **Age** | 1.02 | 1.00 | 1.02 |
| **Sex (women)** | 0.46 | 0.40 | 0.53 |
| **BMI (kg/m^2^)** | 1.04 | 1.02 | 1.05 |
| **Smoking status (never)** | 1 (ref.) |  |  |
| **Smoking status (current smoker)** | 0.73 | 0.61 | 0.87 |
| **Smoking status (former)** | 1.05 | 0.90 | 1.23 |
| **Physical activity (x 100 MET-min/day)** | 1.02 | 1.00 | 1.05 |
| **Level education (primary or illiterate)** | 1 (ref.) |  |  |
| **Level education (secondary)** | 0.78 | 0.67 | 0.91 |
| **Level education (university)** | 0.64 | 0.52 | 0.78 |
| **13-point Score** | 0.95 | 0.92 | 0.98 |
| **Total energy intake (x 1000 Kcal/day)** | 0.71 | 0.64 | 0.79 |

Model 1 was adjusted for: age (years), sex, geographic recruitment area and BMI (kg/m^2^). The abdominal obesity component of the metabolic syndrome was not adjusted by BMI.

Model 2 was additionally adjusted for smoking status (never, former or current smoker), leisure time physical activity (x100 MET-min/day) and level education (primary or illiterate, secondary and university).

Model 3 was additionally adjusted for energy intake (x 1000 kcal/day) and adherence to the Mediterranean diet (13-point score).

Extremes of total energy intake were excluded.
